# Early Prediction of Refractory Epilepsy in Children Under Artificial Intelligence Neural Network

**DOI:** 10.3389/fnbot.2021.690220

**Published:** 2021-06-17

**Authors:** Yueyan Huang, Qingfeng Li, Qian Yang, Zhijing Huang, Hongbo Gao, Yunan Xu, Lianghua Liao

**Affiliations:** ^1^Department of Pediatrics, Affiliated Hospital of Youjiang Medical College for Nationalities, Baise, China; ^2^Department of Radiology, Affiliated Hospital of Youjiang Medical College for Nationalities, Baise, China; ^3^Center for Diagnosis and Research of Pathological Diseases, Affiliated Hospital of Youjiang Medical College for Nationalities, Baise, China

**Keywords:** convolutional neural network, refractory epilepsy in children, EEG, MRI, disease prediction

## Abstract

In order to realize the early prediction of refractory epilepsy in children, data preprocessing technology was used to improve the data quality, and the detection model of refractory epilepsy in children based on convolutional neural network (CNN) was established. Then, the data in the epilepsy electroencephalography (EEG) signal public data set was used for model training and the diagnosis of refractory epilepsy in children. Moreover, back propagation neural network (BPNN), support vector machine (SVM), XGBoost, gradient boosting decision tree (GBDT), AdaBoost algorithm were introduced for comparison. The results showed that the early prediction accuracy of BP, SVM, XGBoost, GBDT, AdaBoost, and the algorithm in this study for refractory epilepsy in children were 0.745, 0.778, 0.885, 0.846, 0.874, and 0.941, respectively. The sensitivities were 0.81, 0.826, 0.822, 0.84, 0.859, and 0.918, respectively. The specificities were 0.683, 0.696, 0.743, 0.792, 0.84, and 0.905, respectively. The accuracy was 0.707, 0.732, 0.765, 0.802, 0.839, and 0.881, respectively. The recall rates were 0.69, 0.716, 0.753, 0.784, 0.813, and 0.877, respectively. F1 scores were 0.698, 0.724, 0.759, 0.793, 0.826, and 0.879, respectively. Through the comparisons of the above six indicators, the algorithm proposed in this study was significantly higher than other algorithms, suggesting that the proposed algorithm was more accurate in early prediction of refractory epilepsy in children. Analysis of the EEG characteristics and magnetic resonance imaging (MRI) images of refractory epilepsy in children suggested that the MRI images of patients' brains under this algorithm had obvious characteristics. The reason for the prediction error of the algorithm was that the duration of epilepsy was too short or the EEG of the patient didn't change notably during the epileptic seizure. In summary, the prediction method of refractory epilepsy in children based on CNN was accurate, which had broad adoption prospects in assisting clinicians in the examination and diagnosis of refractory epilepsy in children.

## Introduction

As one of the most common chronic neurological diseases, epilepsy has complex pathological causes and different clinical manifestations, and its incidence is about 0.4–1.0% (Manford, 2017). Epilepsy patients have different causes. Studies found that it may be related to genetic factors, history of febrile convulsions, malformation of cortical development (MCD), injuries during pregnancy and childbirth, gliomas (dysembryoplastic neuropithelial tumor), and cerebrovascular diseases (Sapkota et al., 2018). Epilepsy can cause great harm to patients, causing sudden brain dysfunction, cognitive impairment, depression, and death (Roger et al., 2018). Epilepsy mostly occurs in children or adolescents. After the correct use of antiepileptic drugs (AEDs) and regular treatment, the effective control rate can reach about 70–80%. However, there are still 20–30% of children who have repeated seizures even after regular AEDs treatment and develop drug-refractory epilepsy (Herta and Dorfer, 2019; van der Lende et al., 2019).

Refractory epilepsy is usually defined as the correct application of two anti-epileptic drugs but still has not achieved continuous seizure-free. The treatment methods for refractory epilepsy in children include AEDs, epilepsy surgery, neuromodulation, and ketogenic diet (KD) (Stevelink et al., 2018; Sekar and Pack, 2019). In the treatment of AEDs, appropriate selection should be made according to the type of epileptic seizures to avoid adverse reactions caused by improper medication (Barbella et al., 2018; Lambrechts et al., 2018; Wheless et al., 2018). Electroencephalogram (EEG) is the main basis for the diagnosis of epilepsy. In addition, since computed tomography (CT) and magnetic resonance imaging (MRI) can accurately reflect the brain morphology of patients with epilepsy, they are also widely used in early inspection and surgical replication treatment of refractory epilepsy in children (Qin et al., 2020). However, the early diagnosis of refractory epilepsy mostly relies on the subjective judgment of clinicians. Long-term, high-intensity, and repetitive manual testing will affect the accuracy of clinicians' judgments, resulting in an increase in false detection rates. Moreover, different doctors have different diagnosis results to the same patients, which makes the diagnosis more difficult (Huang et al., 2020). With the development and application of artificial intelligence in the medical field, deep learning has gradually replaced the traditional machine learning technology in the auxiliary diagnosis of clinical diseases (Varatharajah et al., 2018; Guo et al., 2019). To realize the early automatic diagnosis and prediction of refractory epilepsy in children, a new artificial intelligence neural network algorithm was designed based on previous studies, which aimed to realize early brain image recognition and automatic detection of children with refractory epilepsy.

## Materials and Methods

### Data Set

The data used in this study all came from the epilepsy EEG data set and the EEG signal of hospital. In the epilepsy EEG signal data set, each subset contained 100 pieces of 23.6 s in length, and they were single channel EEG with a sampling frequency of 173.61 Hz. The information generated by the hospital EEG dataset was collected from patients with refractory epilepsy monitored 24 h a day without AEDs.

### Prediction of Refractory Epilepsy in Children Based on Convolutional Neural Networks

With the continuous progression of deep learning, disease prediction methods are gradually diversified. Using deep learning algorithms to realize precise diagnosis and prediction of diseases is a hot topic in intelligent medical care. The early prediction model of refractory epilepsy in children in this study includes data preprocessing and convolutional neural network (CNN). In actual forecasting, due to the different nature of the data and different dimensions between each feature data, the data should be standardized and normalized first to reduce the impact of different dimensions on the overall performance. In this study, the maximum and minimum values are normalized to process the data, and the processing is expressed by the following equation.

(1) $$\begin{array}{r} x\;=\;\frac{x_{0}-x_{\min}}{x_{\max}-x_{\min}} \end{array}$$

In Equation (1), *x* represents the data after the maximum and minimum values are normalized, *x*_0_ represents the data before processing, *x*_*max*_ represents the maximum value of the dimensional data, and *x*_*min*_ represents the minimum value of the dimensional data. To improve the training speed, it needs to reduce the dimensionality of the data. The principal component analysis is performed to extract data redundancy information. For a data matrix *H*, it is supposed that it contains *a* samples, and *b* represents the row vector of each sample with different attributes. Then, *H* = (*H*_1_, *H*_2_, *H*_3_, ⋯, *H*_*a*_) where $H_{1}={\left(h_{1}^{1},h_{2}^{1},h_{3}^{1},\cdots ,h_{b}^{1}\right)}^{T}$. In the matrix *H, P* represents the column vector of *H*. Then, $P={\left(P_{1},P_{2},P_{3},\cdots ,P_{b}\right)}^{T}$. Through calculation, the covariance based on different *P* is obtained, and the matrix *G* is formed, as shown in the following equation.

(2) $$\begin{array}{r} G\left(m,n\right)=\text{cov}\left(P_{m},P_{n}\right)\\ \;=E\left[\left(p_{m}-c_{m}\right)*\left(p_{n}+c_{n}\right)\right] \end{array}$$

In Equation (2), *c*_*m*_ and *c*_*n*_ represent the expectation of *P*_*m*_ and *P*_*n*_, respectively. The matrix element values reflect the degree of linear correlation between two random variables. When the covariance is 0, it means that the two random variables are linearly independent. The larger the covariance, the stronger the correlation between the two random variables. When the eigenvalues and eigenvectors of *H* are calculated, *H* is transformed to obtain the transformation matrix of *H*. In the process of data compression, the larger the feature value, the larger the numerical range and variance of the feature. Therefore, the eigenvectors corresponding to the larger eigenvalues should be used to construct the transformation matrix first to obtain new features with higher importance.

After the data preprocessing, CNN is adopted to predict refractory epilepsy in children. Convolutional neural network includes convolutional layer, sampling layer, connection layer, and output layer. The purpose of the convolutional layer is extracting features, and the purpose of the sampling layer is extracting the main special diagnosis, and then the fully connected layer summarizes the features, and uses the classifier to predict and recognize the data. In the convolutional layer, there are multiple neurons sharing weight parameters to form a feature sub-graph, and then multiple feature sub-graphs together form a convolutional layer. Such approach can reduce the links between levels and can avoid overfitting (Wang and Li, 2020). In the operation, the matrix and the convolution kernel are multiplied first, and several convolution units are formed after the operation of the convolution with a step length of *n*. The value of the feature sub-image obtained by convolution is the product of all the convolution kernels and the corresponding weights. In a CNN, an activation function is usually required to determine whether the output of the neuron in each convolutional layer exceeds the threshold. When the feature strength in a certain area is too weak to extract the feature, the output is 0. At this time, other regional features won't affect the convolution operation to extract other features. In general, the activation function is a non-linear function. Common activation functions are Sigmoid function, *Tanh* function, and rectified linear unit (ReLU) function. In this experiment, *Tanh* function is taken as the activation function, and its function expression is as follows.

(3) $$\begin{array}{r} Tanh\left(x\right)=\frac{e^{x}-e^{-x}}{e^{x}+e^{-x}} \end{array}$$

**Figure 1:**
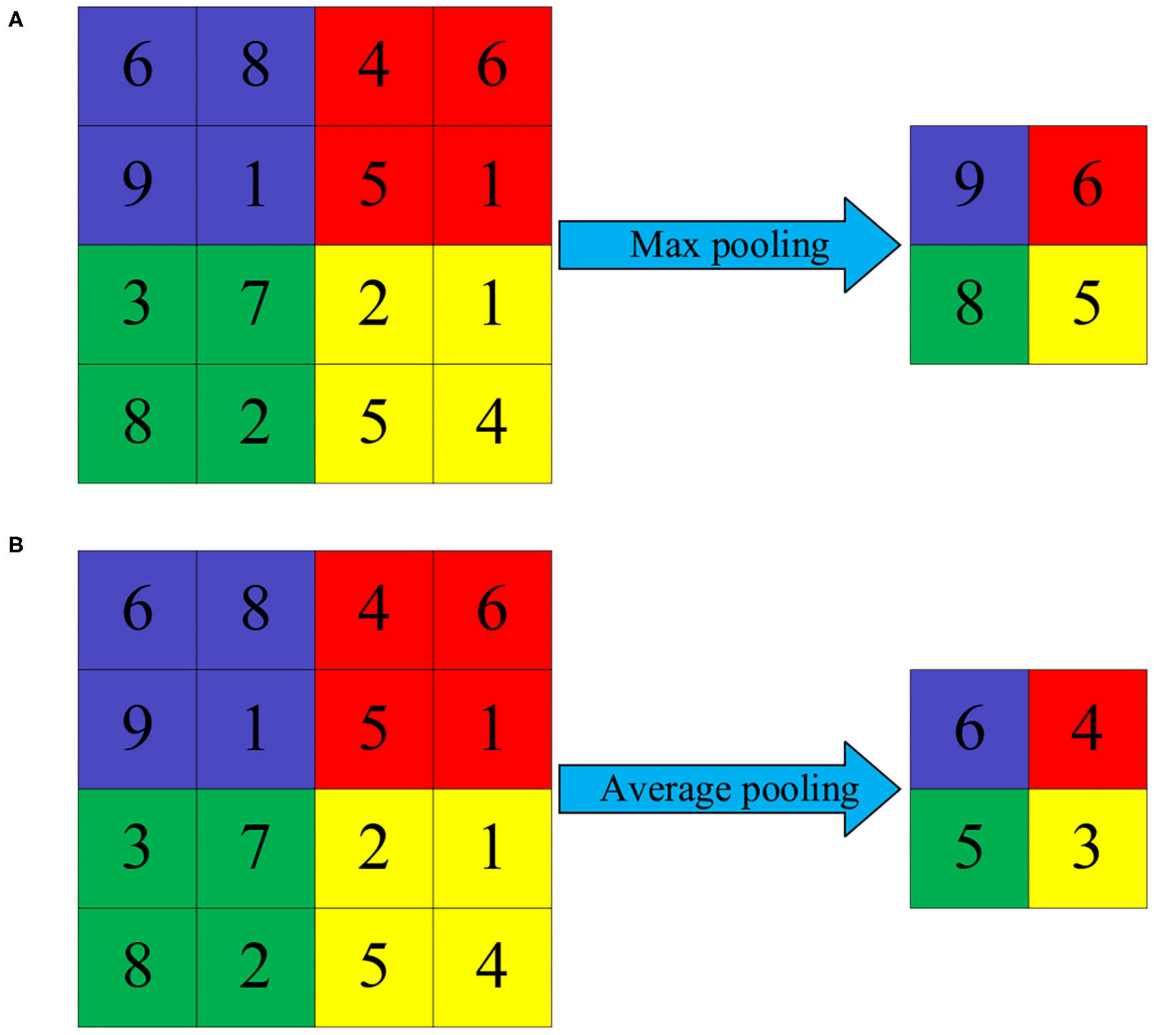
Schematic diagram of pooling operation. **(A)** Maximum pooling and **(B)** average pooling.

The sampling layer is also called the pooling layer. The currently adopted pooling methods include maximum pooling and average pooling. The pooling layer belongs to a special kind of convolution. The purpose of the entire pooling process is reducing the training parameters of the training model and simplifying the training model. Maximum pooling can reduce the error caused by the bias of the estimated mean value caused by the parameter error of the convolutional layer. Average pooling can reduce the error caused by the increase in the variance of the estimated value caused by the limited size of the neighborhood. The two pooling methods aim to well-preserve the image texture and the background information of the image (Türk and Özerdem, 2019). The operation process of maximum pooling is keeping only the maximum value of the feature extracted by the convolutional layer. When the feature doesn't exist or is not obvious, the maximum value can also be extracted. However, the maximum value is very small, and the features extracted by the maximum pooling will remain in the output of the maximized sub-sampling (Figure 1).

**Figure 2:**
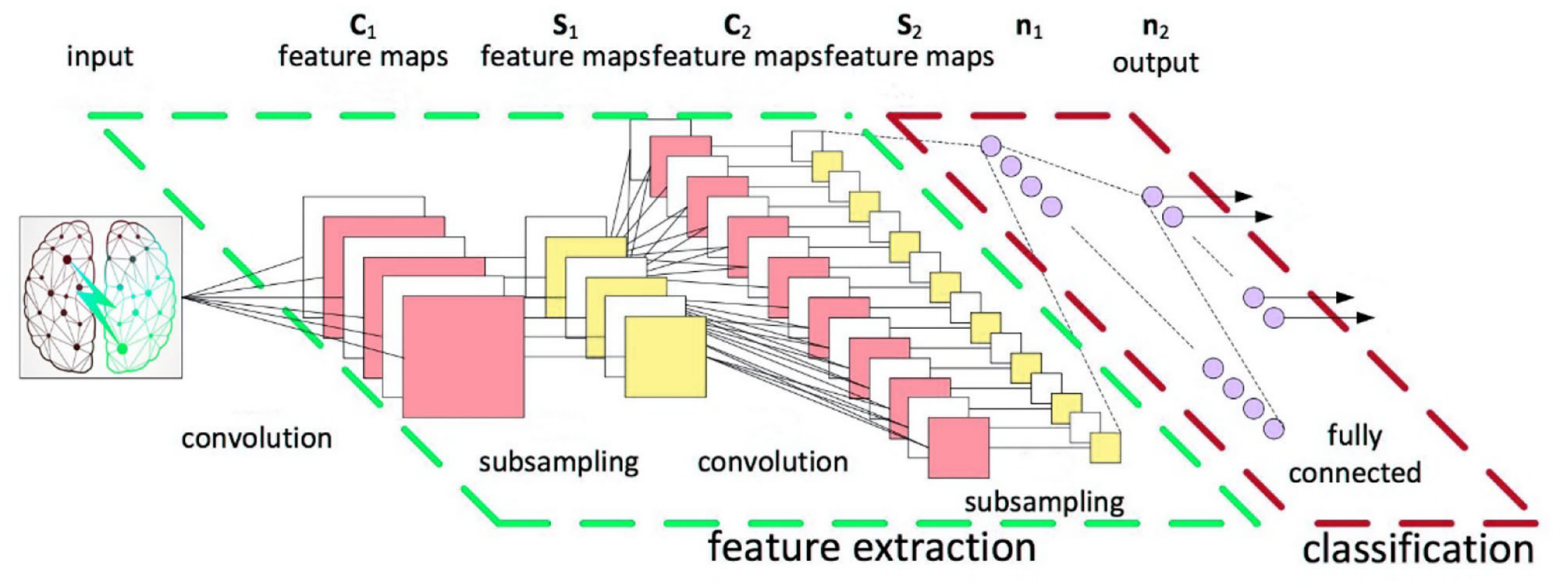
CNN calculation model.

After the original data extracts features through the convolutional layer and main features from the sub-sampling layer, the image information is transmitted to the output layer through the fully connected layer. The purpose of the fully connected layer is mapping the image information processed by the convolutional layer and the sub-sampling layer into the label space mapped to the sample. The fully connected layer processes the image feature information, transforming the two-dimensional feature map into a one-dimensional feature vector. Then, after summarizing the feature information, it performs full neural network training. Different from the convolutional layer, the fully connected layer captures the non-linear relationship between the comprehensive feature information of the convolutional layer and the sub-sampling layer to learn feature information, so as to accurately classify features (Figure 2).

To prevent the model from overfitting due to the excessive feature information contained in the fully connected layer during the CNN calculation process, it is usually necessary to introduce a normalization function for the final classification. In this study, the Softmax function is used as the classification function of the final classification layer. The function expression is as follows.

(4) $$\begin{array}{r} \sigma \left(i\right)=\frac{e^{i}}{\overset{K}{\underset{K=1}{\sum }}e^{j}} \end{array}$$

In Equation (3), σ represents the probability of samples in a category, *i* represents the output of the neuron, and *K* represents the number of categories.

### CNN Training

The artificial intelligence-based CNN is employed to build a high-precision classification model through the distribution of training data. In this model, each training sample is composed of an input data and a data label. The purpose of CNN is constructing a network model through the distribution and characteristics of data to realize the inference function of refractory epilepsy in children. Its essence is inferring the prediction results of unknown samples through a mathematical model constructed by the training and classification results of multiple known models. Since whether each sample suffers from refractory epilepsy is known in the early model training process, a CNN detection model about whether the patient has refractory epilepsy in children can be constructed by supervised learning to predict whether the patient has refractory epilepsy in children.

Tensorflow and Keras are utilized as the development framework of the model. The programming interface of Keras is adopted based on Tensorflow, and the open-source data set is used to train the model. After preprocessing of the data feature combination, the cross-validation method is adopted to evaluate the performance of the model authenticating.

The model parameters are set for testing. The size of the convolution kernel in the C1 convolution layer is 6 × 6, which contains 24 convolution kernels, and the step size is set to 1. The size of the convolution kernel in the C2 convolution layer is 6 × 6, including 20 convolution kernels, and the step size is set to 1. The activation function uses the *Tanh* function, the epoch is set to 100 times, the mini-batch size is 30, and the learning rate is 0.2. Maximum pooling is performed to sample the maximum value of the 12 × 12 feature matrix obtained after two layers of convolution. The mapping gets a 6 × 6 matrix, the number of convolution kernels is 20, and the step size is 1. *Tanh* function is connected for non-linear mapping. After the second 2D convolution operation, the size of the convolution kernel is 3 × 3, the number of convolution kernels is 24, and the step size is still 1. After the convolution operation, the maximum pooling operation is used, and the pooling step is set to 2. The 2D feature map after the maximum pooling is stretched into a one-dimensional vector as the input of the fully connected layer. The number of neurons is set to 844 in the second fully connected layer, and then the *Tanh* non-linear mapping is connected to again. To prevent the model from overfitting due to the excessive feature information contained in the fully connected layer during the CNN calculation process, the Softmax function is introduced as the classification function of the final classification layer. In this study, the patient's brain signal was analyzed in a 5 s period. First, the patient's third, fourth, and fifth layer wavelet reconstruction sub-band signals were formed into a two-dimensional distribution to represent electrical signals. Then, the convolutional layer and the pooling layer of the CNN extracted the characteristics of each segment of the patient's EEG signal. The extracted features were transmitted to the classifier through the fully connected layer for judgment, and finally the early prediction results of refractory epilepsy in children were obtained.

### CNN Evaluation

To evaluate the data model established, the prediction performance of CNN is evaluated regarding the accuracy, sensitivity, specificity, and F1 score. The calculations are as follows.

(5) $$\begin{array}{r} Acc=\frac{TP+TN}{TP+TN+FP+FN} \end{array}$$

(6) $$\begin{array}{r} Sen=\frac{TP}{TP+FN} \end{array}$$

(7) $$\begin{array}{r} Spe=\frac{TN}{TN+FP} \end{array}$$

In Equations (5)–(7), *Acc* represents accuracy, *Sen* represents sensitivity, and *Spe* represents specificity. *TP* represents the number of positive samples that are correctly classified, *TN* represents the number of negative samples that are correctly classified, *FP* represents the number of negative samples that are incorrectly classified, and *FN* represents the number of positive samples that are misclassified. In practice, the accuracy and recall of different scenarios need to be measured. The calculations are as follows.

(8) $$\begin{array}{r} Pre=\frac{TP}{FP+TP} \end{array}$$

(9) $$\begin{array}{r} Rec=\frac{TP}{FN+TP} \end{array}$$

Then, F1 score is expressed as follows.

(10) $$\begin{array}{r} F1=2\times \frac{Pre\times Rec}{Pre+Rec} \end{array}$$

In Equations (8)–(10), *Pre* represents the precision and *Rec* represents the recall. The F1 score is a weighted average of the model precision and the recall, and its value range is 0–1.

### Statistical Analysis

SPSS 19.0 was employed to perform statistical analysis on the data. The count data were expressed in %, and the χ^2^-test was utilized. The *t*-test was adopted between two independent samples. *P* < 0.05 indicated that the difference was statistically significant.

## Experimental Results

### EEG Analysis of Refractory Epilepsy in Children Based on CNN

**Figure 3:**
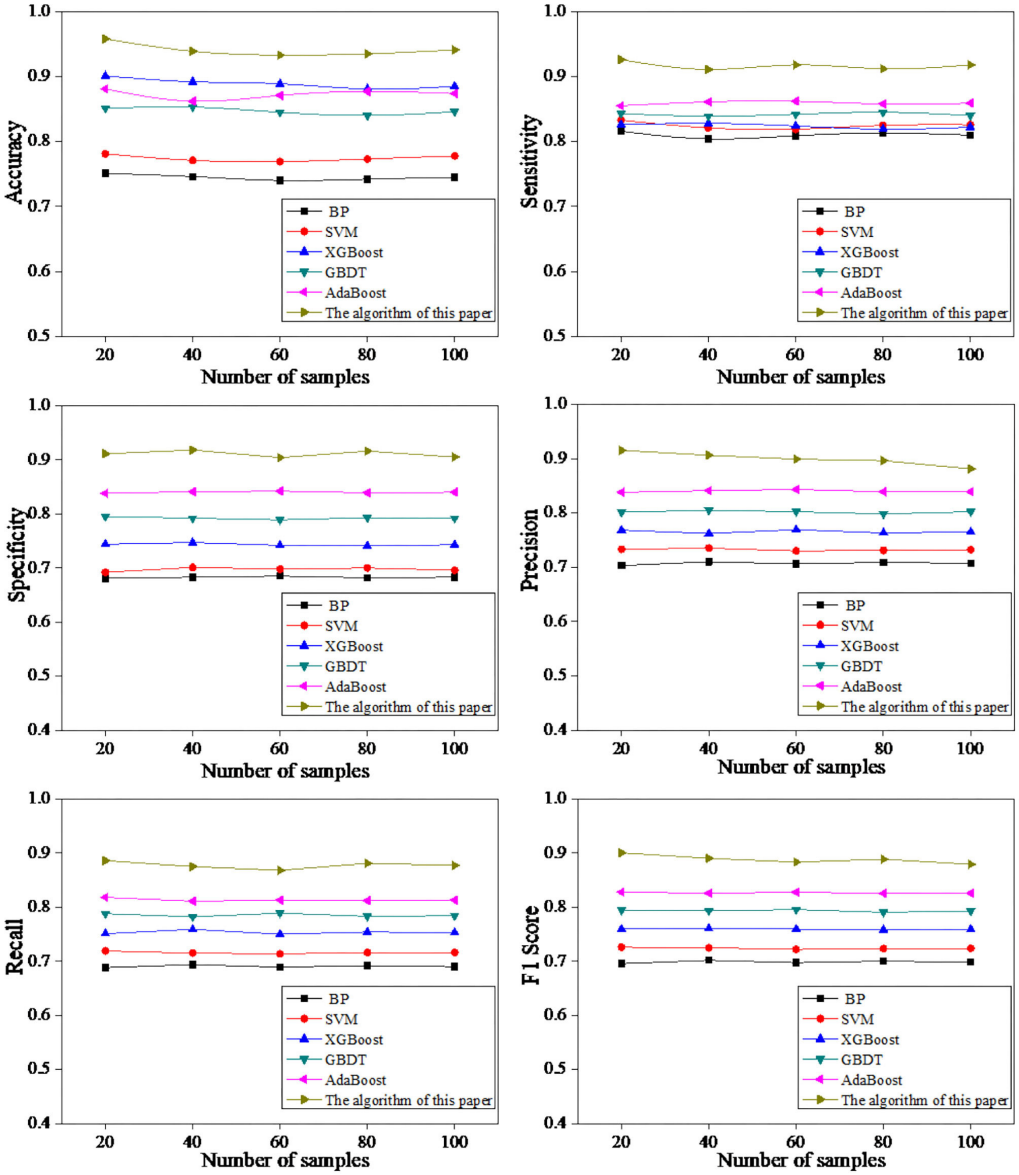
Comparison of prediction effects based on different algorithms.

In this study, the patient's brain signals were input into the CNN prediction model, and the detection results of the brain electrical signals of children with refractory epilepsy were obtained. The detection results were shown in Figure 3. The detection results based on different algorithms were compared, and it was found that when the number of samples in the training set was 20, the early prediction accuracy of the proposed algorithm for refractory epilepsy in children was 0.958. The sensitivity was 0.926, the specificity was 0.911, and the accuracy was 0.915, the recall was 0.886, and the F1 score was 0.900. When the number of samples in the training set was 40, the early prediction accuracy of this algorithm for refractory epilepsy in children was 0.939, the sensitivity was 0.911, the specificity was 0.918, the precision was 0.906, the recall was 0.875, and the F1 score was 0.890. When the number of samples in the training set was 60, the early prediction accuracy of this algorithm for refractory epilepsy in children was 0.933, the sensitivity was 0.918, the specificity was 0.904, the precision was 0.899, the recall was 0.868, and the F1 score was 0.883. When the number of samples in the training set was 80, the early prediction accuracy of this algorithm for refractory epilepsy in children was 0.935, the sensitivity was 0.912, the specificity was 0.916, the precision was 0.896, the recall was 0.881, and the F1 score was 0.888. When the number of training set samples was 100, the early prediction accuracy of this algorithm for refractory epilepsy in children was 0.941, sensitivity was 0.918, specificity was 0.905, precision was 0.881, recall was 0.877, and F1 score was 0.879. It was found that the accuracy, sensitivity, specificity, precision, recall, and F1 score of the proposed algorithm for early prediction of refractory epilepsy in children were greatly higher than other algorithms, and the differences were considerable.

**Figure 4:**
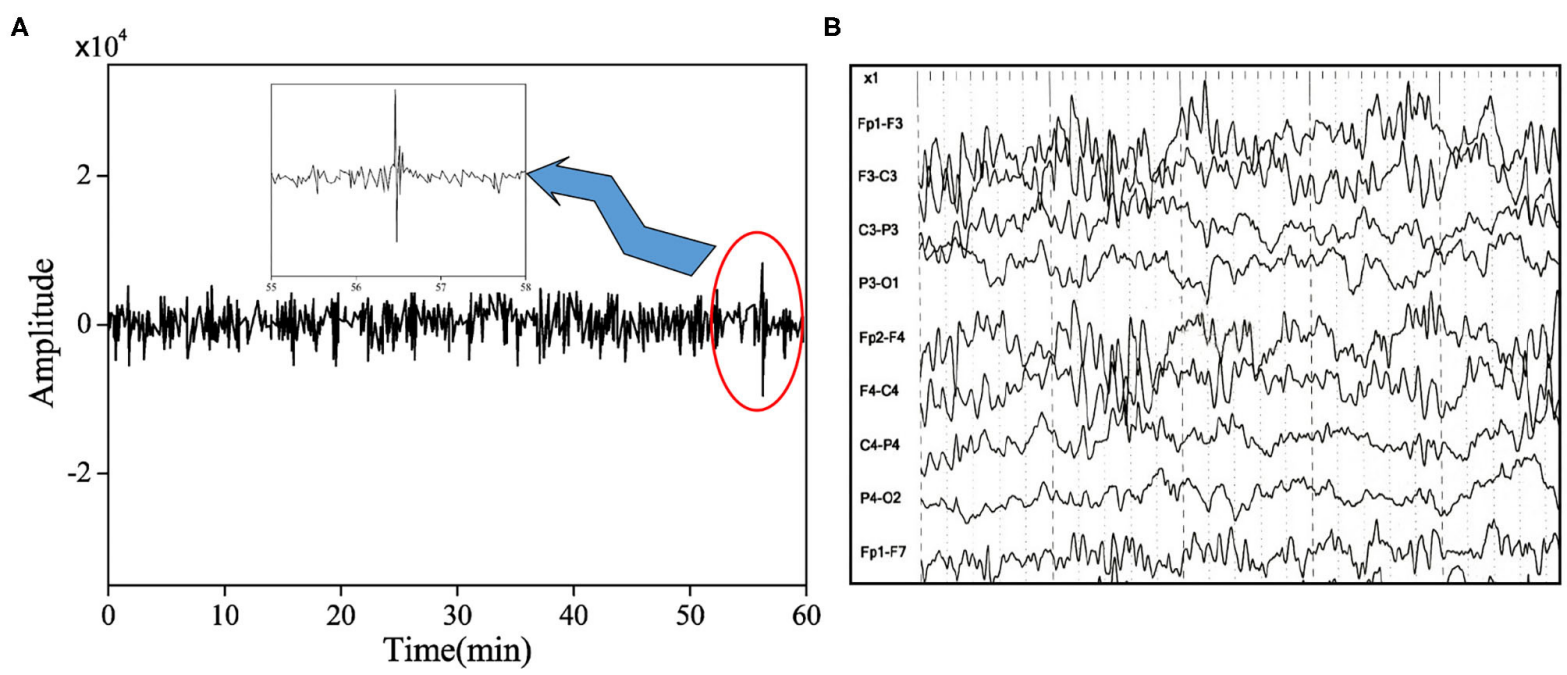
EEG signal of refractory epilepsy in children during seizure. **(A)** The EEG signal of the undetected epileptic patient and **(B)** the EEG signal of the patient during the epileptic seizure.

Electroencephalography feature detection was performed on refractory epilepsy in children. The detected epileptic seizures had obvious changes in EEG characteristics and had a long duration (Figure 4). When CNN was employed to detect the EEG of patients, it didn't detect epileptic seizures in some patients. After the cause was analyzed, it was found that the duration of epilepsy in such patients was very short (<10 s). Therefore, when the EEG was smoothed, it was filtered out by the instrument by mistake. In addition, in some cases, no significant changes in the patient's EEG were detected during epileptic seizures. Therefore, the features extracted by the CNN algorithm were not significant, resulting in no seizures detected in patients.

### Brain MRI Image Analysis of Refractory Epilepsy in Children Based on CNN

**Figure 5:**
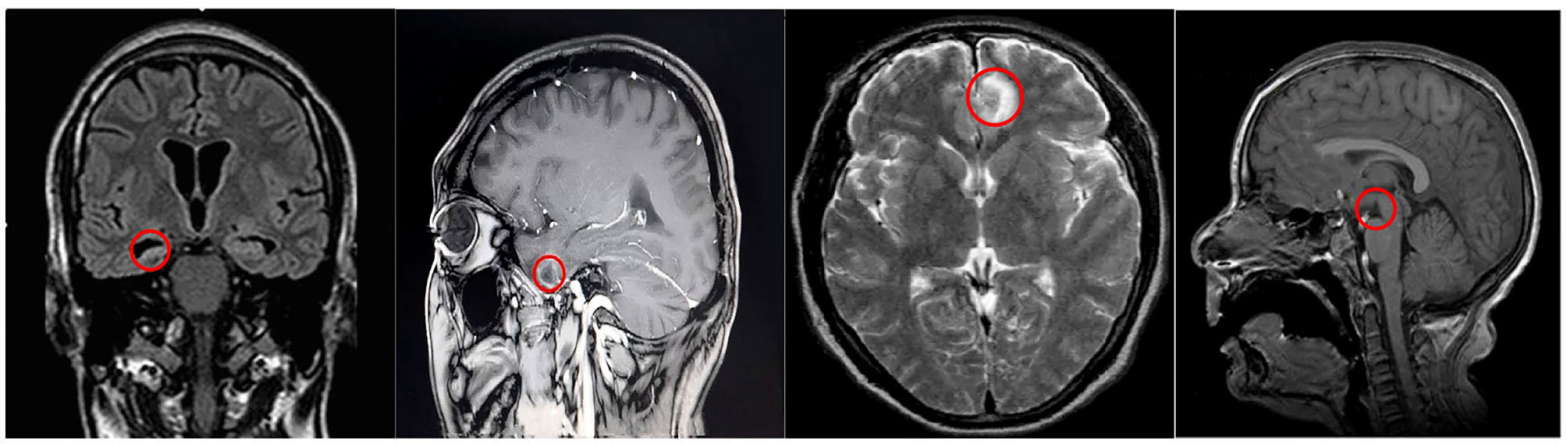
MRI images of refractory epilepsy in children brain.

Before accurate segmentation of refractory epilepsy in children brain MRI images, brain tissue needed to be extracted in advance. Before CNN was employed to register the image, the image was preprocessed to improve the accuracy of the CNN algorithm once. The calculation result of the algorithm was shown in Figure 5. The results showed that the brain MRI images of patients with refractory epilepsy in children showed obvious hippocampal atrophy, structural abnormalities, and enlargement of the temporal horn, atrophy of the temporal lobe, and changes in the white matter of the front temporal lobe. The above results suggested that the proposed algorithm had favorable diagnostic effect on brain MRI images of refractory epilepsy in children.

## Discussion

Refractory epilepsy is difficult to treat, easy to relapse, and prone to status epilepticus, which seriously affects the patient's quality of life. Moreover, the fatality rate and disability rate of patients are high, which has caused great distress to patients (Cai et al., 2017; Gaspard et al., 2018). Refractory epilepsy exhibits drug resistance and is difficult to treat. Clinical surgery is considered to be an effective method for the treatment of refractory epilepsy. However, it is necessary to evaluate and locate the patient's lesions in advance (Høgsbro-Rode et al., 2017). Studies showed that refractory epilepsy in children was usually accompanied by abnormal changes in EEG (von Lehe and Parpaley, 2017). Therefore, the early stage of brainwave imaging of pediatric patients is beneficial to the diagnosis and treatment of refractory epilepsy in children.

In this study, the open-source dataset of refractory epilepsy in children was utilized to train the CNN proposed, and the data was preprocessed first. Then, the image was output through a series of operations such as convolution, pooling, and full connection. After which, the early prediction effect of the CNN model on refractory epilepsy in children was analyzed, and other models were introduced for comparison. The results showed that when the training set was 100, the early prediction accuracy of this algorithm for refractory epilepsy in children was 0.941, the sensitivity was 0.918, the specificity was 0.905, the precision was 0.881, the recall was 0.877, and the F1 score was 0.879. The five indicators were greatly higher than other algorithms, suggesting that the algorithm proposed had a better predictive effect, which was similar to the results of Liu et al. (2020). Analysis of the EEG signals of non-detected patients found that some patients had no obvious changes in their EEGs during epileptic seizures, or the duration of epilepsy was short (<10 s). As a result, it was mistakenly filtered out when the algorithm smoothed the filtering, which affected the accuracy of diagnosis. Finally, the brain MRI images of patients with refractory epilepsy in children were analyzed, and the results showed that the brain features of the patients were obvious, suggesting that the algorithm proposed had a certain diagnostic effect on MRI images of epilepsy.

## Conclusion

In this study, the CNN algorithm was employed to realize the early prediction of refractory epilepsy in children. The results showed that after data preprocessing, CNN can predict and diagnose early refractory epilepsy in children accurately, and had a favorable effect on MRI image processing of the patient's brain. This algorithm has high guiding significance in the early diagnosis and treatment of refractory epilepsy in children, and it is worthy of clinical adoption. However, this work doesn't subdivide the types of refractory epilepsy in children, and doesn't evaluate the specific effects of the proposed algorithm to assist in the treatment. These shortcomings require more in-depth research with more data in future work.

## Data Availability Statement

The original contributions presented in the study are included in the article/supplementary material, further inquiries can be directed to the corresponding author/s.

## Author Contributions

YH: writing—original draft and conceptualization. QL: writing—review and editing and methodology. QY: data curation and software. ZH: supervision and resources. HG: formal analysis. YX: validation. LL: visualization. All authors contributed to the article and approved the submitted version.

## Conflict of Interest

The authors declare that the research was conducted in the absence of any commercial or financial relationships that could be construed as a potential conflict of interest.
